# The Potential Utility of Predicted One Bond Carbon-Proton Coupling Constants in the Structure Elucidation of Small Organic Molecules by NMR Spectroscopy

**DOI:** 10.1371/journal.pone.0111576

**Published:** 2014-11-03

**Authors:** Chandrasekhar Venkata, Mark J. Forster, Peter W. A. Howe, Christoph Steinbeck

**Affiliations:** 1 European Bioinformatics Institute, Wellcome Trust Genome Campus, Hinxton, Cambridge, United Kingdom; 2 Chemical Research, Syngenta, Jealott's Hill Research Centre, Bracknell, United Kingdom; 3 Product Safety, Syngenta, Jealott's Hill Research Centre, Bracknell, United Kingdom; University of Calgary, Canada

## Abstract

NMR spectroscopy is the most popular technique used for structure elucidation of small organic molecules in solution, but incorrect structures are regularly reported. One-bond proton-carbon J-couplings provide additional information about chemical structure because they are determined by different features of molecular structure than are proton and carbon chemical shifts. However, these couplings are not routinely used to validate proposed structures because few software tools exist to predict them. This study assesses the accuracy of Density Functional Theory for predicting them using 396 published experimental observations from a diverse range of small organic molecules. With the B3LYP functional and the TZVP basis set, Density Functional Theory calculations using the open-source software package NWChem can predict one-bond CH J-couplings with good accuracy for most classes of small organic molecule. The root-mean-square deviation after correction is 1.5 Hz for most *sp^3^* CH pairs and 1.9 Hz for *sp^2^* pairs; larger errors are observed for *sp^3^* pairs with multiple electronegative substituents and for *sp* pairs. These results suggest that prediction of one-bond CH J-couplings by Density Functional Theory is sufficiently accurate for structure validation. This will be of particular use in strained ring systems and heterocycles which have characteristic couplings and which pose challenges for structure elucidation.

## Background

NMR (Nuclear Magnetic Resonance) spectroscopy remains the most popular method of determining both the covalent structure and conformation of small organic molecules in solution, owing to the detailed chemical information that can be obtained from NMR spectra acquired on sub-milligramme amounts of material [1]. Proton and ^13^C chemical shifts provide information about the chemical environment of atoms, proton-proton and proton-carbon J-couplings provide information about the connectivity between atoms, and proton integrals provide information about the multiplicity of groups of atoms. These principal sources of information can be supplemented by ^15^N chemical shifts, by nuclear Overhauser effect (nOe) or rotating frame Overhauser effect (rOe) data, and by proton-carbon residual dipolar couplings where a sufficient amount of the substance is available. These different pieces of information can be interpreted by experienced scientists, or by automated structure determination software, to determine the structure of an unknown molecule [2].

Given the range of information that can be obtained from NMR spectra, it may seem surprising that incorrect structures are regularly published in the literature [3], [4]. Many of these are errors in relative configuration, but there are still numerous cases where the core of the molecular skeleton is incorrect. It is too simplistic to blame incorrect structures on misinterpretation or lack of experience. To asses structures, experts and automated structure determination programs compare predicted chemical shifts of possible structures to those observed in spectra. Carbon chemical shifts can be predicted with reasonable accuracy using literature data or *ab initio* using Kohn-Sham Density Functional Theory (DFT) methods while prediction of proton shifts has much lower accuracy owing to their dependence on solvent and through-space interactions such as hydrogen bonding and ring current effects [5]. In many cases, several possible structures are consistent with the observed chemical shifts so choosing which is correct can become an exercise of judgement involving knowledge of the synthetic scheme or biosynthetic pathway, interpretation of through-space information from nOe or rOe spectra, or consideration of the magnitude of long-range proton-carbon J-couplings.

**Figure 1:**
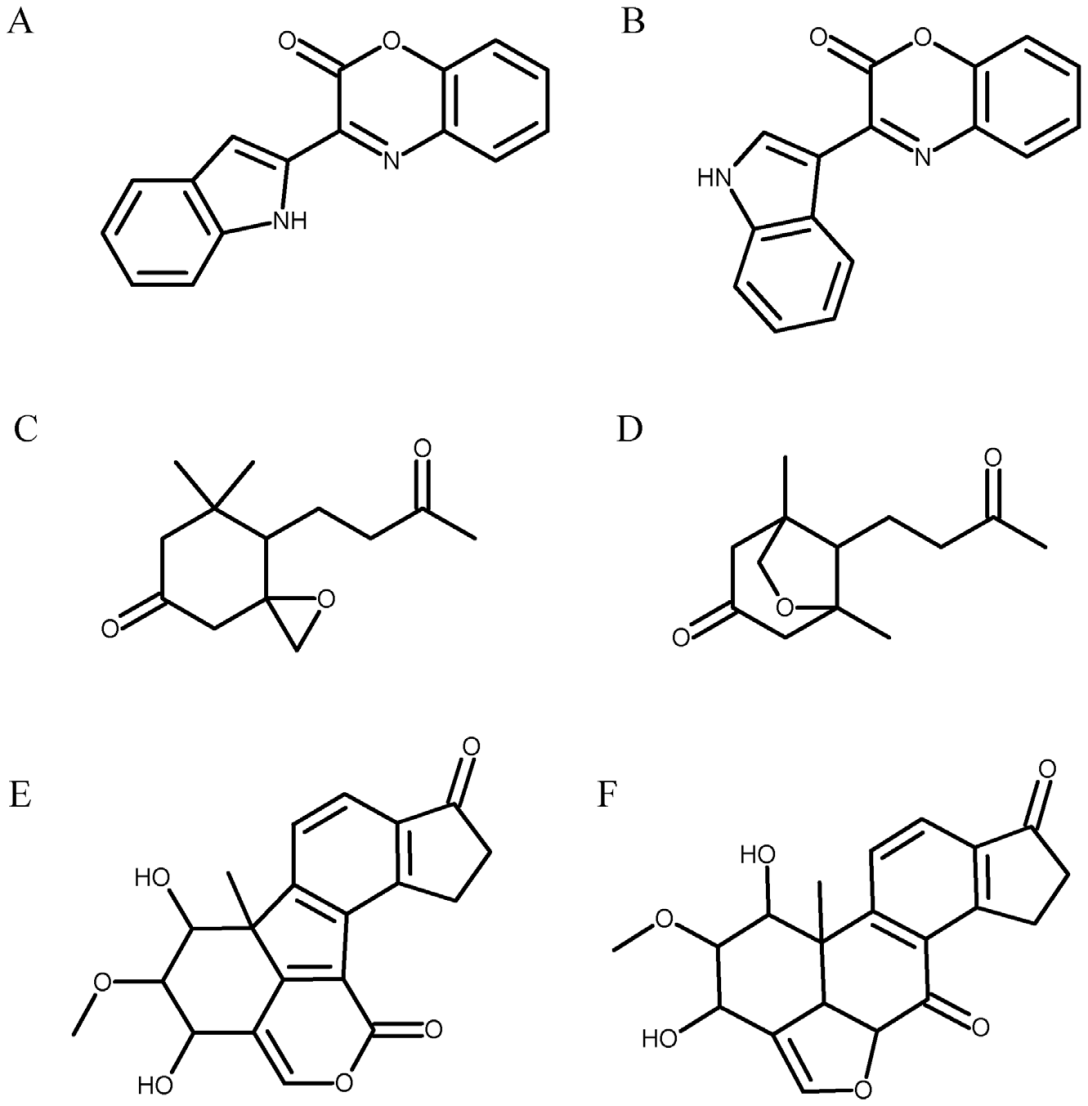
Structures of natural products challenging for NMR spectroscopy. A and B: Two of the candidate structures of Cephalandole A evaluated by Gross *et al.* B is the correct structure (6). The proposed (C) and revised structures (D) of TAEMC161 [7], [8]. The proposed (E) and revised structures (F) of Annuionone A [9], [10].

This paper proposes that an additional NMR observable, ^1^J_CH_, can now be predicted with sufficient accuracy to help in the structure determination process. It is generally believed that ^1^J_CH_ is highly correlated with carbon chemical shift, but this correlation breaks down in a significant number of cases because it is determined by different atomic properties from the carbon chemical shift. The main properties that determine ^1^J_CH_ are the degree of *s* character of the CH bond and the electronegativity of other substituents on the carbon atom. In contrast, carbon chemical shift differences are typically determined by the paramagnetic shielding term, which is in turn is strongly influenced by the asymmetric distribution of the *2p* atomic orbitals around the carbon nucleus as well as the presence of low energy separation excited electronic states [5]. These differences mean that ^1^J_CH_ values are especially useful for identifying aromatic heterocycles (such as thiophenes and azoles), alkynes and strained ring systems (such as epoxides and norbornanes) which often cause difficulties in structure determination. Heterocycles contain few protons, reducing the number of long-range proton-carbon correlations that can be observed and can include oxygen and sulphur atoms which do not give useful solution-state NMR spectra. Bridged ring systems such as norbornanes result in complicated long range proton-carbon correlations owing to the numbers of atoms two, three and four few bonds apart, and can be further complicated by unusual dihedral angles causing unusual long-range ^1^H, ^13^C J-couplings. Figure 1 shows three examples of structure determinations illustrate structures of this type where ^1^J_CH_ information could have been useful. In the case of Cephalandole A, Gross *et al* [6] found chemical shifts as inconclusive and used atomic force microscopy to distinguish between 4 proposed structures but two of these could have been eliminated by measuring the ^1^J_CH_ of the indole singlet proton. If the proton were in the 2 position (Figure 1 A), its ^1^J_CH_ would be close to 175 Hz, while if the proton were in the 1 position (Figure 1 B), it would be close to 185 Hz. A second example is that of TAEMC161. The ^1^J_CH_ of the lactone heterocycle methine originally proposed [7] (Figure 1 C) would be approximately 195 Hz, while that of the correct Viridiol structure deduced by comparison with other natural products [8] (Figure 1 D) would be over 200 Hz. A final example is Annuionone A where the epoxide methylene protons in the initially proposed structure [9] (Figure 1 E) would be expected to have ^1^J_CH_ of over 170 Hz, while the equivalent protons in the correct 3,2,1 bridged ether structure [10] (Figure 1 F) would be about 160 Hz.

^1^J_CH_ can be measured with similar sensitivity to carbon chemical shifts using ^13^C satellites in proton observed spectra [5], from peak splitting in carbon spectra or by quantitative J-correlation [11], [12]. Despite this ease of measurement, and the clear utility of ^1^J_CH_, they are rarely used for structure determination or verification because few NMR spectroscopists are familiar with typical values of ^1^J_CH_, and there are no easy-to-use software packages for predicting them. Simple empirical calculation methods have been proposed for predicting them in substituted methanes [13] and aromatics [14], in substituted alkanes based on substituent electronegativity and bond length [15] and in strained rings based on bond angles [16]. Unfortunately, these cannot be combined with one another and no empirical methods are available for *sp* or aromatic heterocycle atoms. Several collections of experimental ^1^J_CH_ have been published [17], [18], but no software has been developed to use them for prediction.

The alternative approaches for calculating ^1^J_CH_ are *ab initio* quantum mechanical methods. Because ^1^J_CH_ depends largely on the Fermi contact term, initial pioneering work on the application of *ab initio* methods by Pople and co workers [19] demonstrated good accuracy, and in more recent times DFT has since become the method of choice [20]. These methods approximate the exact electron density which determines J-couplings from a single determinant reference wave function created for a fictitious system of non-interacting electrons. For J-couplings, best results are obtained using functionals that combine the Generalized Gradient Approximation (GGA) with exact Hartree-Fock exchange. The main drawback of DFT methods is that they are computationally expensive and therefore slow compared to empirical methods based on library searching.

Several recent papers have shown that DFT can accurately calculate ^1^J_CH_. Maximoff *et al*. [21] demonstrated the potential of the method while subsequent work by Helgaker *et al* [20] suggested that it is important to carry out geometry optimization and ^1^J_CH_ calculation using the same basis set and functional. San Fabian *et al* [22] investigated 35 combinations of functionals and basis sets using 88 experimental ^1^J_CH_ values from 68 molecules, and concluded that standard deviations of approximately 10 Hz could be obtained. Linear regression of observed versus calculated couplings reduced the standard deviation to less than 5 Hz, with a total range of errors of approximately 25 Hz. Their reported quality metrics were similar for many of the basis sets and functionals they tested, but they recommended the functionals PBE, B3P86 and B97-2 and basis sets Hill-su3, aug-ccp-VTZ-J and pcJ-2 with the note that the commonly used combination of B3LYP functional and TZVP basis set gave similar results and had the advantage of being much less computationally expensive. A standard deviation of less than 5 Hz was very encouraging because solvent effects were not considered and can alter ^1^J_CH_ by a few Hertz [23].

This paper extends the work of San Fabian *et al* by comparing DFT predictions of ^1^J_CH_ couplings with experimental observations for more than 200 molecules including strained rings such as norbornane and cyclopropane and heterocycles such as tetrazole and pyridine which were not addressed by the work of San Fabian *et al* (of the 200 molecules, 29 were also investigated by San Fabian *et al*). The combination of B3LYP and TZVP was chosen for faster computation and to ensure widespread availability. One of several open-source quantum chemistry packages, NWChem [24], was used to ensure that the methods reported here are freely available to all researchers. Software tools were developed to extract and visualise the calculated couplings and cases of large deviations between experimental and calculated values were investigated in more detail. The results confirm that DFT has good accuracy for predicting ^1^J_CH_ couplings for most types of CH pairs so are of potential use in structures elucidation.

## Materials and Methods

Chemical structures and corresponding observed couplings were compiled from the website of University of Wisconsin [18] and from the textbook of Kalinowski *et al.* [25]. The Chemical structures were redrawn manually and saved in the CML (Chemical Markup Language) format, which were subsequently converted into input files for NWChem using OpenBabel [26]. Geometry Optimzation and DFT calculations used NWChem version 6.3 compiled on the compute cluster at the European Bioinformatics Institute which operates using a modified version of Redhat (Linux OS). The basis set TZVP [27] and the B3LYP functional [28] were used as provided by the standard NWChem distribution. Minimized structure atomic coordinates and couplings were extracted from the NWChem log files using Python scripts. The scripts convert the data in the log file into XML using JUMBO convertors [29] and then the required information is extracted; these scripts and a example NWChem input file are included in Supporting Information. A JAVA program using the CDK library [30] was then used to convert the extracted coordinates to CML files, with J-couplings stored as the corresponding hydrogen atomic property (Information S1). The program also identifies equivalent carbon atoms with multiple attached protons, and averaged the couplings to give a single value for each group (none of the molecules contained a chiral centre so non-equivalence was not an issue). To simplify data analysis and comparison of observed and calculated ^1^JCH values, an HTML5 JavaScript-only web app was developed (Information S7). This visualization application, integrated with JSMol [31] visualizes the molecular structure with the corresponding NWChem calculated and observed ^1^JCH values as the hydrogen atomic properties/labels.

Calculated ^1^JCH values were categorized into 3 separate sets basing on the hybridization of the carbon involved in the C–H coupling. For each category, linear regression was performed between the observed and calculated ^1^JCH using the program R (R function lm). Outliers were identified using the Cook's distance [32] which measures how far, on average, predicted *y*-values will move if the observation in question were dropped from the data set. Data points with Cook's distances greater than 4/(n−k−1) (where *n* is the total number of data points and *k* is the number of predictor variables) and which diverged from the overall pattern were considered as outliers.

NMR spectra were acquired on three molecules to verify previously reported experimental ^1^JCH: 1- and 2- napthaldehyde and fluorobenzene. Samples were obtained from Syngenta's chemical reagent store and dissolved in deuterochloroform. Couplings were measured as the average of the splitting in a proton observed spectrum (either 1D proton or coupled 2D Heteronuclear Single Quantum Correlation spectrum), and that in a carbon observed coupled DEPT-90 (Distortionless Enhanced Polarisation Transfer) spectrum all acquired on a Varian Inova 600 MHz spectrometer fitted with a 5 mm H{CN} cold probe equipped with pulsed-field gradients. Data were acquired, processed and analysed using the spectrometer operating software VNMRJ3.2A (Agilent Inc, Palo Alto CA). The measured couplings for the two napthaldehydes agreed well with literature values, while that for fluorobenzene was 162 Hz, as reported by Reich and not 156 Hz as used by San Fabian *et al* (who did note it might be incorrect).

## Results and Discussion

**Figure 2:**
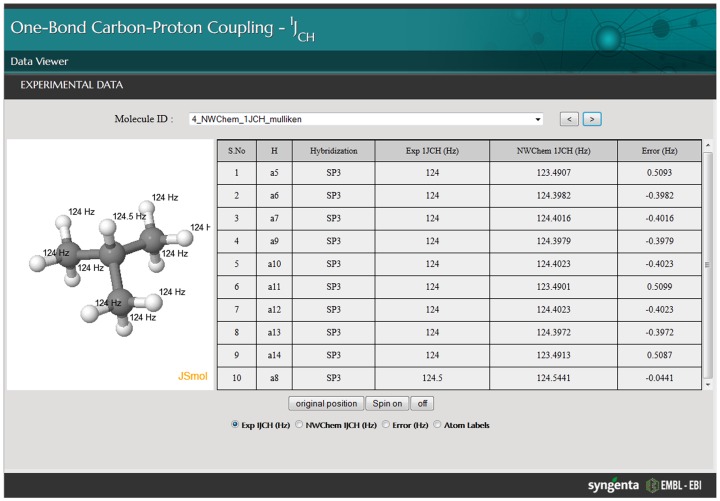
Screenshot of the Javascript application developed to visualise experimental and calculated ^1^J_CH_.

**Figure 3:**
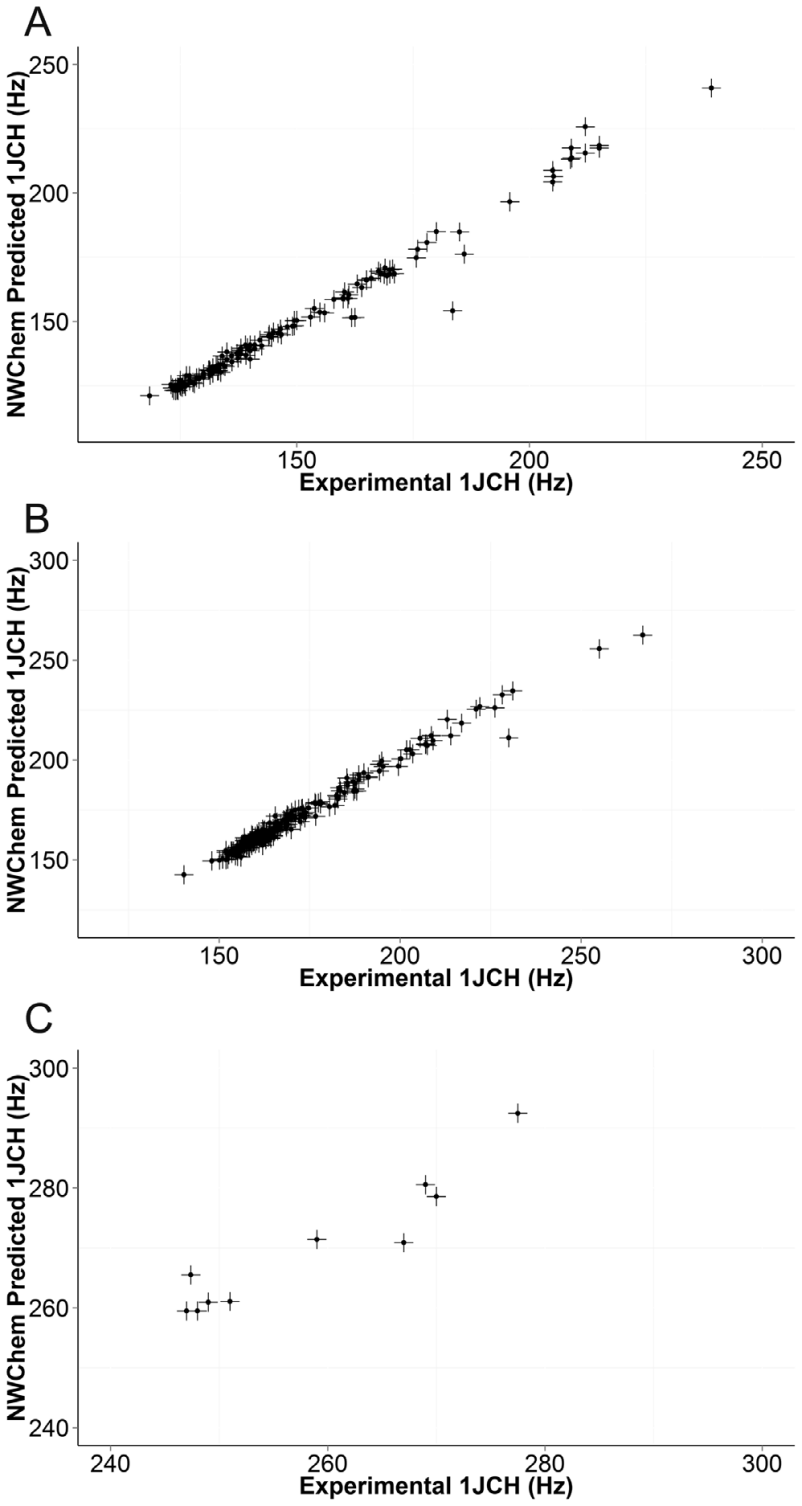
Plots of the observed and predicted ^1^J_CH_ for different hybridization states. A: *sp^3^* pairs. B: *sp^2^* pairs. C: *sp* pairs.

**Figure 4:**
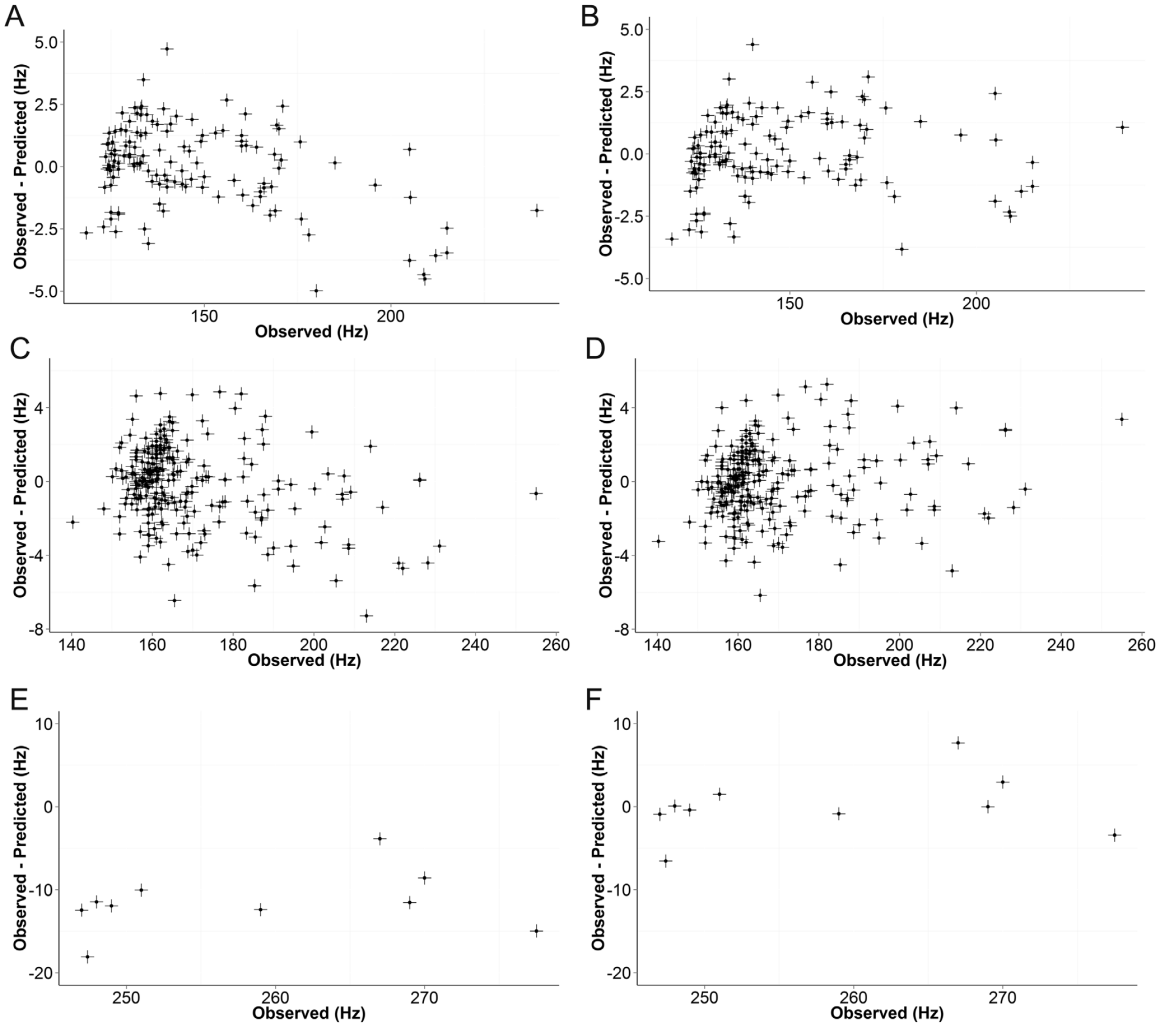
Plots of the difference between observed and predicted versus observed ^1^J_CH_ for different hybridization states. A: uncorrected *sp^3^* pairs. B: *sp^3^* pairs corrected by linear regression. C: uncorrected *sp^2^* pairs. D: sp^2^ pairs corrected by linear regression. E: uncorrected *sp^3^* pairs. F: *sp^3^* pairs corrected by linear regression. Couplings identified as outliers are not shown.

Figure 2 shows a screen-shot of the Javascript application used to visualise the results. This allows the structure and the observed and predicted coupling constants to be easily identified. Figure 3 shows scatter plots of observed versus predicted ^1^J_CH_ for the three hybridisation states before and after linear regression. The agreement is excellent, so Figure 4 shows plots of the difference between observed and predicted ^1^J_CH_ versus observed ^1^J_CH_ for the three hybridisation states before and after linear regression. Table 1 summarises the statistics of the results and demonstrates that DFT predictions agreed extremely well with experimental observations. For both *sp^3^* and *sp^2^* atoms, the standard deviation was less than 2 Hz after removal of outliers (see below) and, after correction by linear regression, all calculated results were within 7 Hz of observations. Accuracy for *sp* atoms was lower and the number of observations was smaller.

**Table 1:** Summary of DFT prediction accuracy for different hybridisation states.

	Total	Outliers	σ	RMSD	Range	σ (corrected)	RMSD	Range (corrected)	*a*	*b*
*sp^3^*	133	6	1.659	1.652	4.72 … –4.99	1.480	1.474	3.83 … –4.39	4.372	0.970
*sp^2^*	253	2	2.075	2.076	7.29 … –4.86	1.909	1.905	6.17 … –5.27	7.400	0.955
*sp*	10	None	3.747	12.071	−3.86 … –18.09	3.747	3.554	7.67 … –6.56	−11.535	1

Shown are the total number of couplings predicted in each hybridisation state: the number classified as outliers: σ (the standard deviation of the difference between the observed and predicted couplings), the RMSD (root-mean square deviation) and the total range of the difference both without and with correction by linear regression: and the intercept (a) and gradient (b) of the linear regression equation. Ranges and standard deviations are reported after exclusion of outliers. Outlier detection was not possible for sp atoms owing to the small data set.

It had been decided to group the observations based on hybridisation states because we expected that the different geometries of electron orbitals would be approximated to different extents by the chosen DFT functional and basis set and therefore linear regression would give different results. The results showed that, in fact, there was very little difference for *sp^2^* and *sp^3^* CH pairs; the two regression equations differ by less than 1 Hz across the entire range so it may have been appropriate that to have grouped the two sets. In contrast the equation for *sp* CH pairs differs from the other two equations by over 10 Hz across the range, suggesting that different factors underlie the difference between observed and predicted couplings.

**Figure 5:**
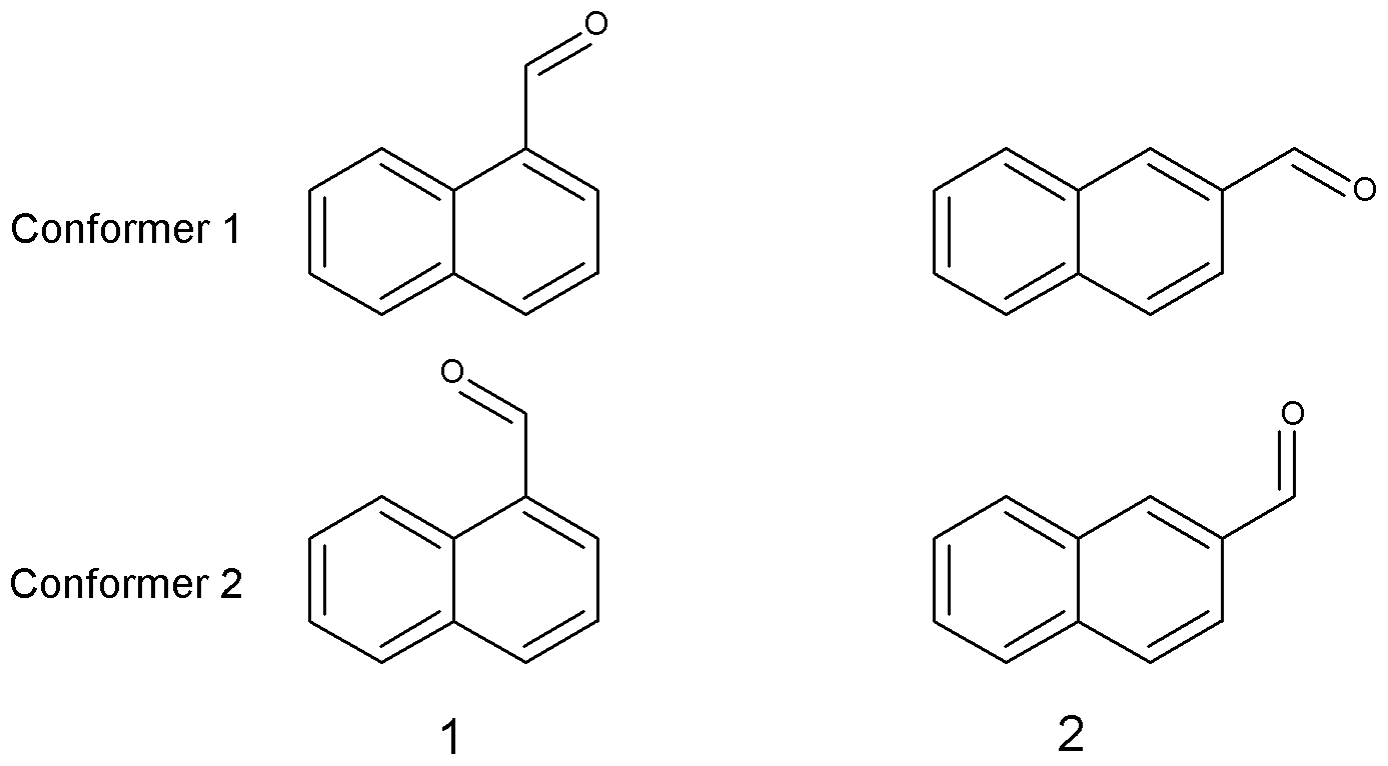
Two conformers of 1-napthaldehyde (1) and 2-napthaldehyde (2).

All outliers were examined and the reported experimental results were checked in the literature where possible. This identified two cases where structures had been incorrectly drawn or atoms had been mis-assigned, in which cases the calculations were repeated with correct data. A third case was CH_2_  =  NCH_3_, where the observed coupling is an average of the methylene and methine couplings owing to rapid tautomerisation of the double bond, while DFT calculated different couplings for the two groups. A fourth interesting example were 1- napthaldehyde (**1**) and 2-napthaldehyde (**2**) (See Figure 5) where the orientation of the carbonyl group was found to have a significant effect on the couplings of neighbouring protons. In the conformer with the carbonyl close to the proton, the coupling calculated by DFT was 6–10 Hz greater than in the conformer with the aldehyde proton close to it (see Table 2). The reported experimental values are close to the average of the calculated values for the two conformers, suggesting that they are averaged in solution. This unexpected finding shows that conformation must be correctly modelled when DFT calculations are undertaken.

**Table 2:** Observed and predicted ^1^J_CH_ couplings for 1- and 2-napthaldehyde in the conformers shown in Figure 3.

Molecule	Atoms	Observed	Predicted (conformer 1)	Predicted (conformer 2)	Average predicted
**1**	C2	157 (158.5)	164.8	157.4	161.1
**1**	C9	164 (162.7)	158.2	169.7	163.9
**2**	C1	159 (159.0)	157.1	163.9	160.5
**2**	C3	164 (163.0)	166.1	158.1	162.2
**2**	C9	160 (160.3)	159.5	160.6	160.1

The bracketed observed couplings are those made in this work.

**Figure 6:**
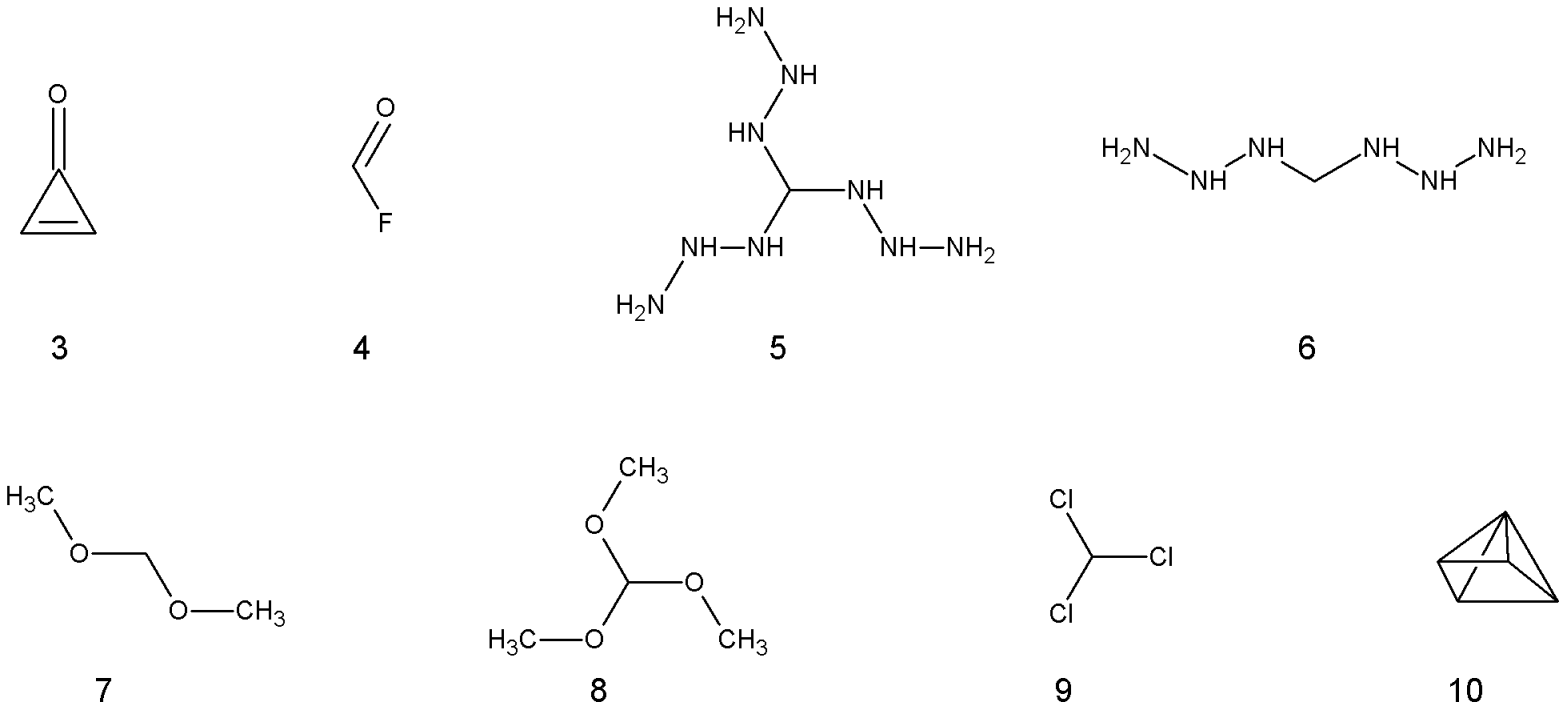
Chemical structures of the 8 molecules identified as outliers following linear regression.

Figure 6 shows the structures of the remaining outliers while Table 3 shows their observed and predicted couplings. We have divided the outliers into three groups for discussion. The first group contains two unusual *sp^2^* hybridised methines, cyclopropenone (**3**) and fluoraldehyde (**4**). Cyclopropenone is known to tautomerise to a zwitterion, so may not have been adequately modelled by DFT of a single tautomer. Fluoraldehyde may be an outlier because it was the *sp^2^* CH with the largest coupling; San Fabian *et al* also reported a 13.5 Hz difference between the observed and predicted coupling for fluoraldehyde. The second group were five methanes substituted with electronegative atoms including chloroform (**5**), trimethoxymethane (**6**) and two methanes with complex azide substituents (**7,8,9**). Some other molecules with electronegative substituents such as CHF_3_ were not outliers so the reason for the large difference between observed and calculated couplings was unclear. The results of San Fabian *et al* include three other methanes with multiple electronegative substituents that were not analysed here (CH_2_(CN)_2_), FH_2_CCN and H_2_CF_2_) and they observed deviations of over 7 Hz between observed and predicted couplings. Note that none of the other combinations of basis set and functional recommended by San Fabian *et al* gave significantly better results for such atoms. The only other *sp^3^* outlier is **10**, a single highly-strained molecule. Other strained rings are accurately modelled by DFT so this may be due to an error, quite possibly in our interpretation of the structure owing to its exceptional nature.

**Table 3:** Molecules containing *sp* or *sp^2^* atoms identified as outliers to the linear regression equations.

Molecule	Observed	Predicted	Difference
**3**	267	262.5	4.5
**4**	230	211.1	18.9
**5**	183.5	154.1	29.4
**6**	162.5	151.6	10.9
**7**	161.8	151.5	10.3
**8**	186	176.2	9.8
**9**	209	217.5	−8.5
**10**	212	225.8	−13.8

One potential cause of differences between observed and predicted couplings is the effect of solvent mentioned in the introduction. The few systematic studies of the relationship between solvent dielectric and ^1^J_CH_ which have been published [23], [33], [34] only considered a narrow range of molecules and reported very different dependencies for different structures (for example, ^1^J_CH_ of chloroform is 8 Hz higher when dissolved in dimethylformamide than when dissolved in cyclohexane, while ^1^J_CH_ of *trans* dichloroethene is only 2 Hz higher). For *sp^3^* CH pairs, solvent dependence can be accurately simulated by DFT [23], [34] but other hybridization states have not been studied. This lack of experimental evidence and theoretical understanding may limit the likely accuracy of ^1^J_CH_ prediction, and deserves further investigation before further effort is made to improve predictions.

Overall, the results obtained here agree well with those of San Fabian *et al* who found that DFT with B3LYP and TZVP accurately predicted couplings for most *sp^3^* and *sp^2^* atoms, with standard deviations of less than 2.1 and 3.2 Hz respectively. As in this work, they found that errors for *sp* atoms were larger with a standard deviation of 4.2 Hz and also obtained larger errors for *sp^3^* carbons substituted with multiple electronegative groups. These results suggest that DFT calculations of couplings are accurate enough for use in structure elucidation, including for heterocycles and bridged ring systems which often pose the largest problems for structure determination. The only functional groups where significant errors were observed are *sp* atoms and *sp^3^* carbon atoms with multiple electronegative substituents, which rarely pose problems in structure elucidation. The main disadvantage of DFT is its speed with coupling calculations taking several hours per molecule. This speed implies that in practice, it should only be used when a small number of consistent structures have been identified and comparison of observed and calculated chemical shifts cannot distinguish which is correct.

## Conclusions

The results presented here show that DFT with the B3LYP functional and the TZVP basis set allows prediction of ^1^J_CH_ with a standard deviation of less than 4 Hz for a wide range of molecules if the results are corrected by linear regression and possible tautomers and conformers are considered. Accuracy is poorer for *sp^3^* CH pairs with multiple electronegative substituents but in only 4 cases was the error larger than 10 Hz. This accuracy is high enough to be of use in determining the structure of small organic molecules; any proposed structure with a difference of more than 5 Hz (*sp^3^* atoms) or 6 Hz (*sp^2^* atoms) between an observed and calculated ^1^J_CH_ is likely to be incorrect and should be re-examined.

## Supporting Information

Information S1 **CML file with ^1^J_CH_ stored as atomic properties.**
(CML)

Information S2 **Example nwchem input file (Ethane).**
(NW) Click here for additional data file.

Information S3 **R script for linear regression fitting and other statistics.**
(R) Click here for additional data file.

Information S4 **R script for linear regression fitting and other statistics.**
(R) Click here for additional data file.

Information S5 **Python script for JCH Data extraction from NWChem log files.**
(PY) Click here for additional data file.

Information S6 **Python script for co-ordinate extraction from NWChem log files.**
(PY) Click here for additional data file.

Information S7 **Javascript visualization Application.**
(RAR)
